# A perspective on cell proliferation kinetics in the root apical meristem

**DOI:** 10.1093/jxb/erab303

**Published:** 2021-06-22

**Authors:** Bénédicte Desvoyes, Clara Echevarría, Crisanto Gutierrez

**Affiliations:** 1 Centro de Biologia Molecular Severo Ochoa, CSIC-UAM, Nicolas Cabrera 1, Cantoblanco, 28049 Madrid, Spain; 2 University of Antwerp, Belgium

**Keywords:** Arabidopsis, cell cycle, cell proliferation, meristem, plant, root

## Abstract

Organogenesis in plants is primarily postembryonic and relies on a strict balance between cell division and cell expansion. The root is a particularly well-suited model to study cell proliferation in detail since the two processes are spatially and temporally separated for all the different tissues. In addition, the root is amenable to detailed microscopic analysis to identify cells progressing through the cell cycle. While it is clear that cell proliferation activity is restricted to the root apical meristem (RAM), understanding cell proliferation kinetics and identifying its parameters have required much effort over many years. Here, we review the main concepts, experimental settings, and findings aimed at obtaining a detailed knowledge of how cells proliferate within the RAM. The combination of novel tools, experimental strategies, and mathematical models has contributed to our current view of cell proliferation in the RAM. We also discuss several lines of research that need to be explored in the future.

## Teaser

A review providing a perspective on concepts and strategies used to understand cell proliferation kinetics and cell cycle parameters in the root apical meristem.

## Introduction

Roots are the plant organs primarily specialized in nutrient sensing and uptake from the soil, but they have other functions as well, such as gravitropism and anchorage to the ground, among others. It has been recognized that there are three major regions along the longitudinal axis of the root: (i) the tip, also known as the root apical meristem (RAM) or the meristematic zone, where all new cells are produced by cell division, (ii) the elongation zone, where cells have arrested the cell cycle, switched to the endocycle and initiated elongation, and (iii) the differentiation zone, where cells continue to expand up to their final size and acquire their fully differentiated state and function. The endocycle is a crucial stage for plant organogenesis and development (Edgar *et al.*, 2014). It is only mentioned here, since it occurs outside the RAM, and the reader is referred to other contributions on this topic (Takatsuka and Umeda, 2014; Bhosale *et al.*, 2018; Lang and Schnittger, 2020). On the radial axis, the RAM consists of concentrical layers of different cell types. These include the epidermis (the outermost layer), the cortex, the endodermis (crucial to isolate the external layers from the inner region by the Casparian strip), the pericycle, and the vascular tissues. In addition, the gravity-sensing columella cells and the root cap constitute the most distal tissues of the RAM. This description refers to the primary root of Arabidopsis, the main topic of this article, although there are variations in other plant species.

Root growth relies on the production of new cells inside the RAM and anisotropic cell expansion outside the RAM in the elongation and differentiation zones of the root. This organ growth, based on cell production and expansion, mostly occurs in the longitudinal axis. One crucial component of the RAM is a group of cells located at the root tip, close to the columella cells, that constitute the quiescent center (QC; reviewed by Dolan *et al.*, 1993; Dubrovsky and Barlow, 2015). The QC is present in root meristems organized as the ‘closed-type’, where all cell lineages in the root can be connected to one tier of initials, or as the ‘open-type’, in which they share common initials (see Baum *et al.*, 2002, for details). The QC was originally known as the ‘cytogenerative centre’ (Clowes, 1953), a visionary term already pointing to its vital role in the maintenance of the whole root, considered ‘the part of the RAM from which all future tissues are derived’ (Clowes, 1954). Thus, the QC is now referred to as the organizing center. It is formed by cells that very rarely divide and it confers stemness potential to cells in direct contact with it. The stem cells (or initials) produce the different cell types that constitute the root (Scheres, 2007).

**Fig. 1.**
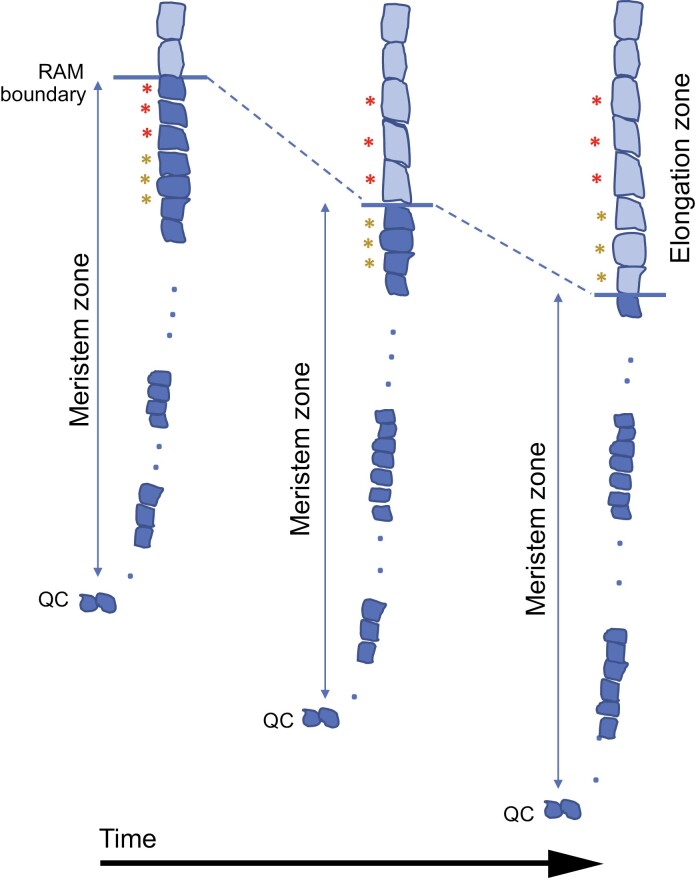
Relative position of RAM cells as a consequence of cell division. Schematic representation of how repeated cell divisions in the RAM (dark blue cells) push the root tip in the rootward direction. Since (i) plant cells remain in the place where they were produced and (ii) the RAM size (from the QC up to the RAM boundary) is maintained pretty constant, once the RAM has been fully developed, production of new cells means that the RAM boundary is displaced in the rootward direction. This means that cells become located at an increasing distance from the QC and, as a consequence, outside the RAM. The effect of root growth by full cell expansion in the elongation (light blue cells) and differentiation zones is not considered for simplicity. Colored asterisks indicate the cells that after each time interval are no longer located inside the RAM. Blue dots indicate an undefined number of meristematic cells in the same file.

Cell division of initials is asymmetric, giving rise to a cell that retains stem cell identity and another that continues undergoing several mitotic cycles, mostly in a transverse (anticlinal) plane (relative to the longitudinal axis of the root), to form the entire cell file in the RAM. Occasionally, division can occur in the longitudinal (periclinal; formative divisions) plane (parallel to the longitudinal axis of the root), originating new cell files in the RAM. Since plant cells remain in the place where they were produced, repeated cell divisions in the RAM push the root tip off, producing cells that become located at an increasing distance from the stem cell location. It is common to use sentences such as ‘RAM cells are displaced and divide until they reach the elongation zone’, which are easy to understand but do not reflect the exact nature of how the root grows and how cells are produced in the RAM (Fig. 1). Thus, root tip growth is better visualized as a tunneling machine that penetrates into the soil or as a growing stalactite in a cave.

Concomitant with cell production in the RAM, specific cell fates are acquired and eventually cells fully differentiate once their position in the root lies outside the RAM due to root tip growth. Therefore, each cell file reflects a developmental trajectory ranging from the more undifferentiated cell, formed after the first asymmetric division of the stem cell, to the early derivatives within the transit amplifying compartment, and finally cells undergoing their last mitotic cycle before entering the endoreplication program. Later, cells reach full differentiation in the differentiated or mature zone of the root. Indeed, differences at the transcriptomic level in the RAM cells have been demonstrated by single-cell RNA-seq, allowing the identification of developmental trajectories of the various cell types in the RAM (Denyer *et al.*, 2019; Jean-Baptiste *et al.*, 2019; Shulse *et al.*, 2019; Ryu *et al.*, 2019; Zhang *et al.*, 2019; Wendrich *et al.*, 2020; Long *et al.*, 2021).

## Strategies to assess cell proliferation in the RAM

The RAM is a complex organ with clear differences along its longitudinal and across its radial axis. As a way to simplify the system, the RAM was considered the sum of single cell files, all of them with the same proliferation properties (López-Sáez *et al.*, 1975; Green and Bauer, 1977; Baskin, 2000). This simplification was, at first, useful to define the time to progress through the entire cell cycle (cell cycle time, *T*), a parameter that relates directly to the cell division rate (1/*T*).

Measurement of cell cycle parameters within the RAM is not trivial. Classic strategies to calculate *T* were based on the determination of the overall cell division rate, deduced from the total cell count in root meristems at different time points. The duration of the mitotic stages (prophase, metaphase, anaphase, and telophase) was originally assessed by their relative frequencies in the total population (Clowes, 1961) and found to be ~3–9 h in different cell types (12 and 200 hours being the extreme cases for the entire cell cycle duration). These studies required tissue disaggregation and, consequently, positional information was lost. Later on, methods were developed that relied on indirect and time-consuming experimental settings, such as labeling with [^3^H]thymidine and measuring the rate of accumulation of metaphases after colchicine treatment (reviewed in Webster, and MacLeod, 1980).

Another experimental strategy for cell cycle time estimation was to follow a given subpopulation of cells through successive mitoses. This population may be composed of radioactively marked cells or polyploid cells, induced by anaphase blocking reagents such as colchicine or caffeine (Van’t Hof, 1966; Giménez-Martín *et al.*, 1966), rendering average cell cycle time values of ~14–16 h.

More recently, continuous treatments with nucleoside analogues, e.g. 5-ethynyl-2′-deoxyuridine (EdU), more friendly than radioactive compounds, have become widespread and the average values obtained for Arabidopsis were ~17–20 h in some reports (Hayashi *et al.*, 2013; Eekhout *et al.*, 2021) and much shorter, ~8–10 h, depending on the cell type, in others (Pasternak *et al.*, 2021, Preprint). While these measurements provide an approximation to the average values of *T* and the cell division rates in different cell types, they fail to be sufficiently reliable when these parameters are to be determined at different positions along the RAM (for a detailed review, see Baskin, 2000). This is a consequence of (i) assuming that all cells in the RAM are actively proliferating (see also below), and (ii) using prolonged labeling treatment periods during which the position of a given cell relative to the QC changes significantly due to division of cells between that position and the QC.

It is true that the use of nucleoside analogues such as EdU has not only simplified enormously the experimental procedures but also avoided potentially deleterious effects on root cells due to radiation from [^3^H]thymidine, particularly when long treatments are carried out. However, it must be also kept in mind that prolonged EdU treatments are not innocuous either. It has been shown that after 6–12 h of incubation at typical concentrations of EdU (2–10 µM) the doubling time of cells in culture increased >35% (Ligasová *et al.*, 2015). Some effects are apparent even after 6 h of continuous EdU incubation, which delays S-phase progression, primarily due to interaction with thymidylate synthase, and leads to G2 arrest (Diermeier-Daucher *et al.*, 2009; Neef and Luedtke, 2011; Ligasová *et al.*, 2015). Therefore, while the use of short pulses with EdU (15–30 min) is the method of choice for labeling S-phase cells, the use of this thymidine analogue for long treatments requires caution. Alternatively, other nucleoside analogues, e.g. (2′*S*)-2′-deoxy-2′-fluoro-5-ethynyluridine (F-*ara*-EdU), seem to be less toxic for the cells (Neef and Luedtke, 2011). However, one major problem with these EdU-labeling strategies (Hayashi *et al.*, 2013) for long periods of time (usually ≥12 h) is that they disregard the increase in the number of labeled cells as a consequence of their passing through mitosis. Thus, considering that after initiating the EdU treatment ~35–40% of cells become labeled, after ~3–4 h (an average G2 duration in Arabidopsis roots) they will start to divide and increase the percentage of EdU-labeled cells not derived from entry of new cells into S-phase.

Alternative strategies have been developed as a way to circumvent problems associated with prolonged treatments with S-phase labeling compounds. Methods based on kinematic approaches were developed to provide a mathematical framework to understand how cell division and expansion contribute to differences in rates of growth at the whole organ level. These methods, mainly applied to root meristems, can be grouped into three classes of approach and the reader is directed to comprehensive reviews for details (Ivanov and Dubrovsky, 1997; Fiorani and Beemster, 2006). Briefly, one group is based on evaluating the rate of cell production, which is inversely proportional to the average cell cycle time. Another group of techniques are based on estimates from the relative frequency of cell flow at a recognizable point of the cell cycle, i.e. metaphase or S-phase, using treatments with colchicine, radioactive thymidine or nucleotide analogues such as EdU. A third group of strategies relies on cycle time estimations by following an identifiable cell population through successive mitoses, e.g. radioactively marked cells (Wimber *et al.*, 1960) or binucleate cells produced by a caffeine pulse (Giménez-Martín *et al.*, 1966). While kinematic approaches provide a systematic method for assessing globally cell proliferation in organs, they make some assumptions: (i) *T* is the same for all meristematic cells, (ii) all meristematic cells proliferate, (iii) the number of cells in a meristem (*N*_m_) or a cell file is constant, and (iv) the flux of cells into and out of the non-proliferating elongation zone is the same. In any case, average *T* values have been deduced with the formula: *T*=(ln2×*N*_m_×*L*_e_)/*V*, where *L*_e_ is the length of fully differentiated cells, relatively easy to measure, *V* is the root growth rate, and *N*_m_ is number of RAM cells normally measured in the cortex layer. This has been done for a large number of plant meristems (Zhukovskaya *et al.*, 2018), rendering a good agreement with the labeling and accumulation strategies. Rymen *et al.* (2010) have discussed kinematic approaches to obtain a general view of average cell proliferation parameters. These studies have served to demonstrate that the relative elemental growth rate in the RAM and elongation zone are spatially separated (van der Weele *et al.*, 2003). Kinematic approaches have also been used to assess cell proliferation kinetics outside the RAM, e.g. for lateral root development (Dowd *et al.*, 2020) and leaves (Rymen *et al.*, 2010). As discussed earlier (Baskin, 2000), whether the cell division rate is constant or varies along the RAM has direct implications for the mechanisms of cell cycle control. Indeed, changes in the cell division rate, should they occur, must rely on specific unknown mechanisms to regulate cell cycle progression of different cell types and in different locations. What the developmental and positional signals are and how they are transduced to the cell cycle machinery to fine tune cell cycle progression is, at present, unknown in molecular terms.

**Fig. 2.**
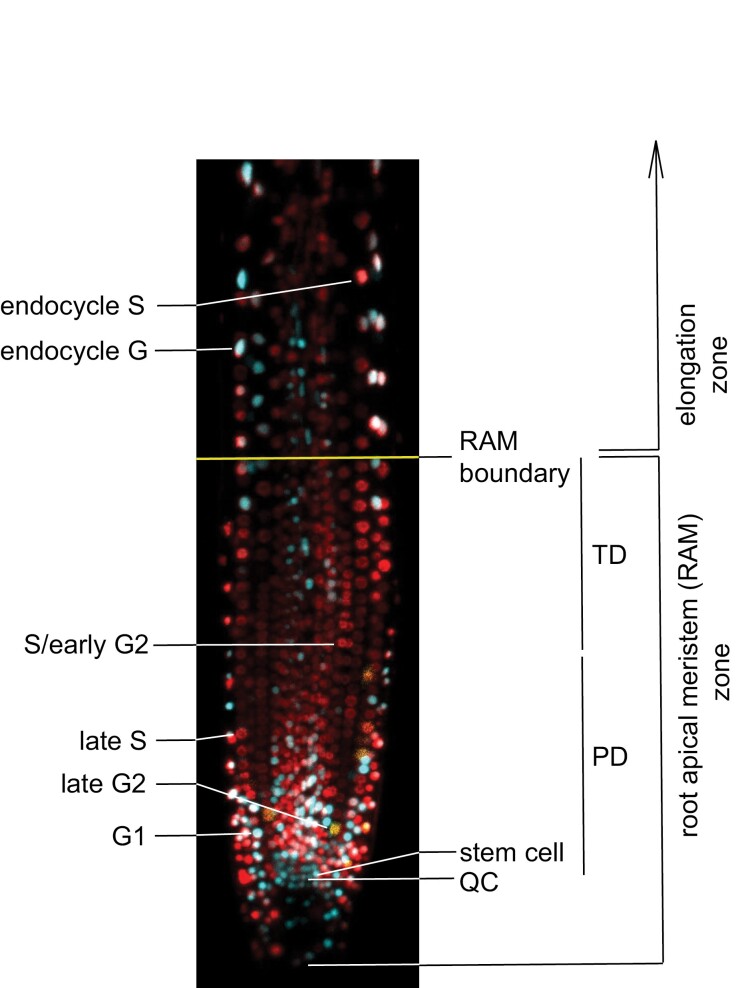
The RAM as visualized with the PlaCCI marker line for cell cycle phase analysis. The two domains of the RAM are included. Nuclei in different phases of mitotic cell cycle and endocycle are indicated. Note that only the beginning of the elongation zone has been included.

In parallel to the development of strategies to assess cell proliferation kinetics in the RAM, efforts have been made to generate tools to identify cycling cells as well as cells in different cell cycle stages. A variety of tools are available and have been comprehensively discussed (Echevarría *et al.*, 2021). Briefly, they rely on labeling with nucleoside analogues or on using plants expressing fluorescent markers. The use of nucleoside analogues dates from the early 1950s where [^3^H]thymidine allowed labeling of S-phase cells (Taylor *et al.*, 1957). Later, non-radioactive analogues were the choice, e.g. 5-bromo-2′-deoxyuridine (BrdU; Xu *et al.*, 1998) or EdU (Salic and Mitchison, 2008; Hayashi *et al.*, 2013). In all these cases, fixed material has to be used, thus providing a static view of the organ under study. The use of plants expressing cell cycle-specific fluorescent markers is the strategy of choice for *in vivo* studies. There are a variety of plant lines expressing constitutive nuclear proteins (Boisnard-Lorig *et al.*, 2001; Otero *et al.*, 2014) and cell cycle phase-specific markers: CYCB1;1 for G2 (Ubeda-Tomás *et al.*, 2009), KNOLLE for cytokinesis (Reichardt *et al.*, 2007), PCNA for identifying cells in S-phase depending on their nuclear pattern (Yokoyama *et al.*, 2016) or a modified histone H4 for S and G2 (Jones *et al.*, 2017). Even more useful are the lines expressing a combination of fluorescent markers. Cytrap (Yin *et al.*, 2014) identifies cells in S+G2 and late G2+M, with reported average cell cycle times of ~16 h. The recently developed PlaCCI line (Desvoyes *et al.*, 2020) allows the identification of cells in G1, S + early G2 and late G2 + mitosis (until anaphase) using a combination of signals produced by three markers expressed under their own promoters, namely CDT1a–CFP, H3.1–mCherry, and CYCB1;1–YFP (Fig. 2). In addition, the three markers and an antibiotic resistance gene are expressed from a single cassette, facilitating its use in genetic crosses of interest. Detailed measurements of cell cycle duration using this tool are not available yet.

## Cell proliferation kinetics: longitudinal zonation of the RAM

As outlined above, a major question is whether all proliferating cells in the RAM possess the same kinetic parameters, e.g. the same total cell cycle and cell cycle phase durations and cell growth rates, independently of their fates and/or positions along the radial and longitudinal axes. Early studies measuring accumulation rates of [^3^H]thymidine-labeled metaphases in maize roots reported that QC and initials had cell cycle durations ranging between 170 h and 240 h (Clowes, 1961). Similar values were confirmed later (Barlow and Macdonald, 1973; Cruz-Ramírez *et al.*, 2013), whereas a scattering of other studies have reported variable cell cycle durations along the RAM (Clowes and Hall, 1962; Mattingly, 1966). The introduction of the concept that QC cells and initials exist in the RAM was probably the first strong reason to think that zonation in the proliferative capacity of cells is a main feature of the RAM (Clowes, 1953; Dubrovsky and Barlow, 2015). It is known that QC and stem cells very rarely divide while the rest of the RAM cells are engaged in active cell divisions and therefore have quite different parameters defining their cell cycle progression. Still, the question remains of whether cell cycle progression occurs at the same rate along the RAM. Since the reports of constancy of the average cell cycle time within the RAM, it has been a common assumption in the field (for a detailed review, see Baskin, 2000) that, except for the initials, the RAM cells that constitute the transit amplifying compartment develop rapid cell cycles of constant duration. Differences in cell cycle duration that have been reported have been ascribed to internal variability of the cell cycle machinery rather than to developmentally regulated differences in cell cycle control or the function of cell cycle factors. It is worth noting that recent *in vivo* measurements over long periods of time, e.g. days, have demonstrated the occurrence of cell cycles of very different durations along the RAM (Rahni and Birnbaum, 2019). Direct measurements have revealed differences in the G2 duration along the RAM (Otero *et al.*, 2016; see below).

A first approach to this question has been the introduction of two conceptual premises: (i) that cell proliferation follows exponential kinetics for all cells in the RAM, i.e. all the cells from the initial up to the last cell before entering the elongation zone, and (ii) that the proliferative fraction (*p*) is between 0 and 1, where *p* is defined as the fraction of total RAM cells (*N*_m_) that are actually cycling (*N*_c_), i.e. *N*_c_/*N*_m_.

In the simplest models used to describe cell proliferation in the RAM, *p* was considered to be 1.0 for the entire RAM, a conclusion based on measurements of cell length along the RAM (for details, see Ivanov and Dubrovsky, 1997). In addition, these models assumed a constant cell cycle time (*T*) and an exponential kinetics for the entire RAM or for most of it. These premises have served to develop methods such as use of the rate of cell production to study cell proliferation kinetics by linking *T*, cell length, and the root growth rate, all measurable parameters from which *T* can be deduced using the already cited equation *T*=(ln2×*N*_m×_*L*_e_)/*V* (Ivanov and Dubrovsky, 1997). However, considering (i) that all cells in the RAM are actually cycling (*N*_m_=*N*_c_, and therefore *p*=1) and (ii) that just beyond the RAM boundary in the transition zone *p*=0, because cells no longer divide, the maintenance of a constant RAM size seems unlikely due to the sharp change in *p* values. In fact, early direct estimations of *p* in several plant species were far from 1 for most of the RAM (Clowes, 1971). A problem, of course, is that so far non-cycling cells cannot be identified. In any case, they should not be randomly distributed in the RAM. If a cell misses one or more cell division cycles at any position in the RAM, symplastic growth imposes the need to compensate this by cell elongation, leading to cells of large size scattered in the RAM and to cell size distributions not observed in real meristems.

Computer simulations have been also used to identify the basic parameters of cell proliferation kinetics in the RAM (Calvo *et al.*, 1982; Pacheco-Escobedo *et al.*, 2016; Rutten and Ten Tusscher, 2019). In these approaches it is possible to evaluate the effects of different values and curves of *p* along the RAM at the cellular level. Although values of *p*=~1 for most of the RAM, with a sharp decrease to *p*=0 at the RAM boundary, have been considered (Ivanov and Dubrovsky, 1997), a high value of *p* up to about half of the RAM and then a progressive decrease up to the RAM boundary renders results closer to real meristems (Calvo *et al.*, 1982). Together, these data led to the proposal of two domains within the RAM (Fig. 2): the proliferation domain (PD) and the transition domain (TD), whereby cells in the PD have a high probability of cycling (*p* close to 1) and cells in the TD possess a decreasing probability of dividing (Ivanov and Dubrovsky, 2013). The boundary between PD and TD has been defined as ‘fuzzy’ by these authors because it occurs at different locations depending on the cell type considered (Baluška and Mancuso, 2013; Ivanov and Dubrovsky, 2013), and is certainly less sharp than the boundary between the RAM and the elongation zone (Lavrekha *et al.*, 2017). In any case, it is a useful boundary to consider since it reflects a key functional difference in cell proliferation potential within the RAM. The use of tailored algorithms, e.g. the multiple structural change algorithm, combined with molecular markers, e.g. CYCB1;1 for G2/M and CCS52A1 for cells entering the endocycle, allows the identification of the PD–TD boundary (Pacheco-Escobedo *et al.*, 2016). Transition from the cell cycle to the endocycle requires a major change in the cellular machinery to eliminate mitosis, e.g. degradation of CYCB by upregulation of CCS52A, among other changes (Edgar *et al.*, 2014; Bhosale *et al.*, 2019; Lang and Schnittger, 2020).

Understanding the RAM with these two domains is valuable because it provides an operative subdivision with functional implications. Indeed, pure exponential kinetics should not be theoretically applied to the entire RAM but only to the PD, where *p*=1. Cell proliferation kinetics in the TD has been described and is not exponential (Calvo *et al.*, 1982). This is in part due to division of cells in the PD that pushes cells located in the TD away from the RAM, something already noticed some time ago (Webster and MacLeod, 1980). In addition, cells located in the TD already receive signals to modify their transcriptional program and trigger the switch to the endocycle (Pacheco-Escobedo *et al.*, 2016; Bhosale *et al.*, 2018).

In spite of this, most estimations of *T* calculate the rate of cell production as *N*_m_×ln2/*T*, assuming exponential kinetics for the whole RAM. However, formally speaking, rate of cell production should be calculated using only the number of cells actually cycling, that is, *N*_c_/*T*, or *N*_m_×*p*/*T*, since *p*=*N*_c_/*N*_m_. However, the similarity between the value of ln2 and the average values of *p* for the whole RAM led to estimations of *T* that are reasonably good (Zhukovskaya *et al.*, 2018), in spite of two oversimplifications (application of exponential proliferation rate for the entire RAM and of constancy of *p*=1 along it). Indeed, differences in cell proliferation kinetics between PD and TD have been considered in estimation of *T*-values (López-Bucio *et al.*, 2014; Napsucialy-Mendivil *et al.*, 2014).

## Outlook

There is compelling evidence that local differences in proliferation potential (or probability) exist within the RAM, e.g. cells in the PD and the TD. However, the question of whether all RAM cells progress through the cell cycle at the same rate is still open. Are there cell location- and/or cell type-specific differences in cell cycle duration? Since the scattering of very early studies >50 years ago indicating differences in T within the RAM, this has not been studied so far in a systematic and comprehensive manner. It has been reported that atrichoblasts and trichoblasts have different cell cycle durations, with trichoblasts developing faster cell cycles, apparently linked to the expression of the *TTG* gene (Berger *et al.*, 1998). Whether these differences in cell cycle progression are due to intrinsic differences in their cell cycle regulation is not yet known. Possible differences in cell cycle phase duration along the RAM have been explored by determining the balance of two histone H3 proteins: the canonical H3.1 is cell cycle regulated and is incorporated during S-phase whereas the H3.3 variant is constitutively expressed and deposited independently of the cell cycle (Probst *et al.*, 2020). By measuring the balance between these two histone H3 proteins, it has been possible to identify the cell population undergoing their last cell cycle, largely located at the end of PD and in the TD (Otero *et al.*, 2016). Furthermore, the G2 phase of these cells is longer than that of cells located in the PD. Whether this cell cycle progression difference is compensated in other cell cycle phases has not yet been explored.

Development of specific microscopic strategies to track dividing cells in the RAM by live-imaging over long periods has revealed important differences in cell cycle duration both in the radial axis (cell types) and in the longitudinal axis (QC, stem cells, proliferating cells). In most cases efforts have been concentrated in the study of QC and stem cells, confirming that they develop long cell cycles (Campilho *et al.*, 2006; Cruz-Ramírez *et al.*, 2013). A major challenge has been to develop experimental settings compatible with live-imaging for a few days to visualize cells directly (Sena *et al.*, 2011; Busch *et al.*, 2012; von Wangenheim *et al.*, 2016, 2017; de Luis Balaguer *et al.*, 2016). Week-long observations of live roots tracking individual cells through consecutive cell cycles have confirmed that QC and stem cells develop long cell cycles but have revealed that cell cycle duration is rapidly reduced in the rest of the RAM (Rahni and Birnbaum, 2019), consistent with the proposal of a gradient in the cell division rate (Rahni *et al.*, 2016). Next, efforts should aim at identifying the molecular basis for such differences and how the cell division pattern is coordinated with the global root growth. The availability of the recently developed tool PlaCCI that serves to identify cell cycle phases (Desvoyes *et al.*, 2020) offers new opportunities to tackle these questions directly.

The developmental zonation of the root, which also reflects zonation of cell cycle kinetics, is established by the complex crosstalk of hormonal signaling pathways. High concentrations of auxin at the root tip are needed to confer proliferation capacity whereas a decreasing gradient is required to counterbalance the cytokinin gradient and define the RAM boundary (Sabatini *et al.*, 1999; Salvi *et al.*, 2020). The auxin gradient parallels that of the PLETHORA (PLT) proteins, maintained by their high stability but diluted over consecutive cell cycles (Galinha *et al.*, 2007; Mähönen *et al.*, 2014). While these appear as the major players in establishing the root zonation, other hormones also play crucial roles, including brassinosteroids, gibberellin, ethylene, and abscisic acid (Moubayidin *et al.*, 2010; Sozzani and Iyer-Pascuzzi, 2014; Thole *et al.*, 2014; Schaller *et al.*, 2014; Jaillais and Vert, 2016; Birnbaum, 2016; Pavelescu *et al.*, 2018; Vaseva *et al.*, 2018; Shimotohno and Scheres, 2019; Vukašinović *et al.*, 2021). How are these gradients of hormonal signals and downstream factors connected with the ability of cells to proliferate? And how is cell cycle regulation coupled to cell growth? These and other questions in this direction remain to be answered in future studies.

We would like to finish by highlighting a couple of sentences taken from published reports several decades ago, referring to the relevance of positional information in regard of cell proliferation potential in the root apical meristem.

‘These properties [related to cell division] may well be influenced by the presence of gradients of growth- and division-regulating substances which are held by some to occur in the root apex … and so the character of the cell cycle may be a function of the position of the cell in the meristem’. (Barlow and Macdonald, 1973)

‘Using positional signal control, it is assumed that any newborn cell located between the beginning of the file and a certain position (Ns) in the cell column is able to start a new division cycle, because it receives a cycling signal, but after the Ns position the cycling cells develop and finish their current cycles without being capable of switching on a new one’. (López-Sáez *et al.*, 1983)

Many aspects still remain to be systematically addressed before we get a complete understanding of how the cell division cycle is regulated along both the radial and the longitudinal axis of the root meristem. The combined use of novel experimental tools and strategies should open exciting avenues in this field.
